# Effects of UBE3A on Cell and Liver Metabolism through the Ubiquitination of PDHA1 and ACAT1

**DOI:** 10.1021/acs.biochem.2c00624

**Published:** 2023-03-15

**Authors:** Kangli Peng, Shirong Wang, Ruochuan Liu, Li Zhou, Geon H. Jeong, In Ho Jeong, Xianpeng Liu, Hiroaki Kiyokawa, Bingzhong Xue, Bo Zhao, Hang Shi, Jun Yin

**Affiliations:** † Engineering Research Center of Cell and Therapeutic Antibody, Ministry of Education, and School of Pharmacy, Shanghai Jiao Tong University, Shanghai 200240, China; ‡ Department of Chemistry, Center for Diagnostics and Therapeutics, Georgia State University, Atlanta, Georgia 30303, United States; § Department of Biology, Georgia State University, Atlanta, Georgia 30303, United States; ∥ Department of Pharmacology, Northwestern University, Chicago, Illinois 60611, United States

## Abstract

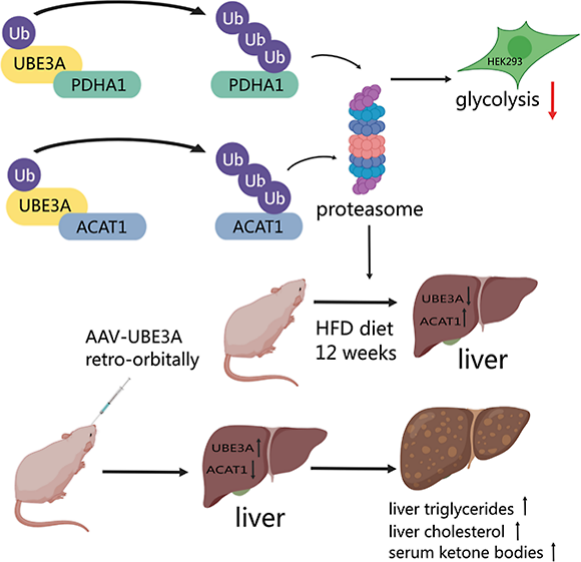

Nonalcoholic fatty liver disease (NAFLD) is substantiated by the reprogramming of liver metabolic pathways that disrupts the homeostasis of lipid and glucose metabolism and thus promotes the progression of the disease. The metabolic pathways associated with NAFLD are regulated at different levels from gene transcription to various post-translational modifications including ubiquitination. Here, we used a novel orthogonal ubiquitin transfer platform to identify pyruvate dehydrogenase A1 (PDHA1) and acetyl-CoA acetyltransferase 1 (ACAT1), two important enzymes that regulate glycolysis and ketogenesis, as substrates of E3 ubiquitin ligase UBE3A/E6AP. We found that overexpression of UBE3A accelerated the degradation of PDHA1 and promoted glycolytic activities in HEK293 cells. Furthermore, a high-fat diet suppressed the expression of UBE3A in the mouse liver, which was associated with increased ACAT1 protein levels, while forced expression of UBE3A in the mouse liver resulted in decreased ACAT1 protein contents. As a result, the mice with forced expression of UBE3A in the liver exhibited enhanced accumulation of triglycerides, cholesterol, and ketone bodies. These results reveal the role of UBE3A in NAFLD development by inducing the degradation of ACAT1 in the liver and promoting lipid storage. Overall, our work uncovers an important mechanism underlying the regulation of glycolysis and lipid metabolism through UBE3A-mediated ubiquitination of PDHA1 and ACAT1 to regulate their stabilities and enzymatic activities in the cell.

## Introduction

The liver is a key organ for maintaining the homeostasis of lipid and glucose metabolism in the human body.^1,2^ The hepatocytes that make up the major biomass of the liver are the bioreactors of essential metabolic pathways, including glycolysis and gluconeogenesis for carbohydrate processing, fatty acid oxidation, ketogenesis, and de novo lipogenesis for lipid metabolism, and the tricarboxylic acid (TCA) cycle for fueling mitochondrial respiration.^3,4^ Precise coordination between different branches of the metabolic pathways in response to the nutritional and physiological cues is a mandate for maintaining proper liver functions.^5,6^ Aberrant activities of hepatic metabolism may induce the development of nonalcoholic fatty liver disease (NAFLD) and later promote its progression toward nonalcoholic steatohepatitis (NASH), cirrhosis, and hepatitis carcinoma.^7−9^ For example, enhanced glycolysis has been found to raise the level of pyruvate and lactate in the blood and the liver,^10,11^ which in turn increases the production of free fatty acids by stimulating the expression of lipogenic enzymes, resulting in the enhanced deposition of triacyl glyceride in the liver of NAFLD patients.^12−14^ Meanwhile, hepatic lipid accumulation promotes fatty acid oxidation and thus increases the production of reactive oxygen species that can cause tissue damage and inflammation in the liver.^15,16^ Furthermore, the increased free fatty acids in NAFLD may prime for hepatic insulin resistance and substantiate the development of hyperglycemia, hyperlipidemia, and type-2 diabetes mellitus (T2DM).^5,17^

Elucidating the underpinning mechanism for regulating hepatic metabolism pathways is instrumental in guiding the development of effective therapies for treating NAFLD. The nutrient metabolism in the liver is regulated at different levels from gene transcription to the control of enzymatic activities by allosteric ligands and post-translational modifications. For one, the transcriptional regulation of metabolic pathways in the liver is exquisitely mediated by a number of hormones such as insulin, glucagon, glucocorticoids, and so forth, which often perform counter-regulatory functions. They cooperate to maintain the proper expression of enzymes and transporters involved in nutrient metabolism via switching on or off signaling cascades that affect the activities of transcriptional factors and the expression of their target genes.^18^ Another regulatory mechanism involves intermediate metabolites such as pyruvate, acetyl-CoA, ATP, and redox factors that may function as allosteric activators or inhibitors to install feedback loops within the metabolic pathways.^19^ In addition, various post-translational modifications such as phosphorylation, acetylation, and *O*-GlcNAcylation impose another layer of control over the metabolic enzymes to match their activities with the cellular demands.^20−23^ Ubiquitination is an abundant protein modification in the cells with the attachment of the 76-residue ubiquitin (UB) to the Lys residues of cellular targets by transferring UB through an enzymatic cascade of UB-activating enzymes (E1), UB-conjugating enzymes (E2), and UB ligases (E3).^24,25^ The first UB attached to the target proteins can be further extended into UB chains of diverse linkages to encode signals regulating the stability and localization of the proteins and their interaction with other cellular partners.^26^ Protein ubiquitination has been well established to regulate critical cellular processes, such as cell cycle, DNA repair, autophagy, and apoptosis.^26−28^ In contrast, the role of protein ubiquitination in regulating the metabolic pathways in the cell has not been extensively studied.

UBE3A, also known as E6AP for E6-associated protein, is an E3 UB ligase implicated in the development of cervical cancer and neurodegeneration such as Angelman syndrome. Due to genomic imprinting, the *Ube3a* gene is paternally inactivated in the brain, so its UB ligase activity is vulnerable to mutations in the maternal copy of the gene that may render the gene defective and cause the delay of neuronal and intellectual development in children.^29,30^ On the other hand, the duplication of the *Ube3a* gene in the maternal chromosome is associated with autism spectrum disorders.^31,32^*Ube3a* is normally expressed from both paternal and maternal copies of the chromosome in the peripheral tissues. The binding of the E6 protein of the human papillomavirus (HPV) with UBE3A would induce the degradation of the tumor suppressor p53, which is causative for the development of cervical cancer.^33,34^ UBE3A has been recently shown to play a role in hepatic steatosis.^35^ In this study, we identified two essential enzymes regulating glycolysis, the TCA cycle, and ketogenesis, namely pyruvate dehydrogenase A1 (PDHA1) and acetyl-CoA acetyltransferase 1 (ACAT1), as ubiquitination targets of UBE3A. We found that UBE3A regulates the stability and enzymatic activity of PDHA1 and ACAT1 in the cell, and the overexpression of UBE3A in the mouse liver would enhance glycolysis and ketogenesis to condition the liver for lipid accumulation as a primer for NAFLD. Our findings unveiled a new axis enabling the regulation of glucose and lipid metabolism by protein ubiquitination and suggested a potential role for UBE3A in NAFLD development.

## Materials and Methods

### Plasmids

The following expression plasmids were used: the pET-28a plasmid for protein expression was from Novagen (Madison, WI, USA). pET-PDHA1 and pET-ACAT1 were generated by the insertion of full-length PDHA1 and ACAT1 genes cloned from HEK293 cDNA into the pET-28a vector. The plvx-IRES-mCherry vector was provided by Feng Qian of Shanghai Jiao Tong University. plvx-UBE3A-IRES was generated by the insertion of the full-length UBE3A gene cloned from HEK293 cDNA into the plvx-IRES-mCherry vector. The pCAG/GFP plasmid (139980) was purchased from Addgene (Cambridge, MA, USA). pCAG-UBE3A was generated by replacing the GFP gene between the BamHI and HindIII restriction sites in the pCAG-GFP vector with the UBE3A gene. The GIPZ non-silencing lentiviral shRNA control (shControl) plasmid (RHS4348) and GIPZ shUBE3A plasmids (#1–6) (RHS4430-200158296, 200161110, 200204991, 200256250, 200264904, 200160626) were purchased from Horizon Discovery (Lafayette, CO, USA).

### Antibodies

The following antibodies were purchased from Santa Cruz Biotechnology (Dallas, TX, USA): anti-PDHA1 (sc-377092), anti-UBE3A (sc-166689), anti-actin (sc-8432), anti-UB (sc-8017), and mouse anti-rabbit IgG-HRP (sc-2357). The goat anti-mouse IgG secondary antibody (31438) was from Thermo Fisher Scientific (Waltham, MA, USA). Anti-ACAT1 (ab168342) was from Abcam (Cambridge, MA, USA).

### Expression and Purification of Recombinant Proteins

*Escherichia coli* BL21 (DE3) cells harboring the expression constructs were cultured at 37 °C and 200 rpm until they reached the exponential phase (OD_600_ = 0.6–0.8) and were induced with 1 mM IPTG at 16 °C for 20 h. Cells were harvested by centrifugation. Cell pellets were resuspended in lysis buffer (50 mM Tris-Base, 500 mM NaCl, 5 mM imidazole, pH 8.0), followed by sonication to complete the lysis process. The lysates were centrifuged, and the supernatant was incubated with Ni-NTA resin (Qiagen, Hilden, German) and rocked gently at 4 °C for 2 h. The protein-bound resin was loaded onto a QIAGEN column, washed twice, and eluted with 250 mM imidazole. Purified proteins were analyzed by SDS-PAGE. The SDS-PAGE gel was stained in a Coomassie R-250 solution for 3 h, followed by distaining.

### In Vitro Ubiquitination

The in vitro ubiquitination assay was performed in 50 μL TBS (137 mM NaCl, 2.7 mM KCl, 24.7 mM Tris-Base, supplemented with 10 mM MgCl_2_ and 1.5 mM ATP, pH 7.4), including 5 μM of substrates PDHA1 (UniProt accession ID: P08559) or ACAT1 (UniProt accession ID: P24752), 1 μM Uba1 (UniProt accession ID: P22314), 5 μM UbcH7 (UniProt accession ID: P68036), 5 μM UBE3A (UniProt accession ID: Q05086), and 20 μM UB (UniProt accession ID: P62975). The reactions were incubated at 37 °C for 2 h, followed by boiling in SDS-PAGE loading buffer. The samples were analyzed by western blotting that was probed with anti-substrate antibodies.

### Cell Culture and Transfection

HEK293 cells were cultured in high-glucose Dulbecco’s modified Eagle medium from Life Technologies (Carlsbad, CA, USA) with 10% fetal bovine serum. Transient transfection of cells was performed with the transfection reagent from Horizon Discovery (T-2006-01, Lafayette, CO, USA), following the manufacturer’s instructions.

### Construction of a Stable shControl Cell Line

Lentiviral particles for the transduction of the shControl plasmid were prepared according to the manufacturer’s protocol for the Trans-Lentiviral shRNA Packaging System. Briefly, the trans-lentiviral packaging plasmid mix (Dharmacon TLP5912) was co-transfected with the shControl plasmid into HEK293T as the packaging cell line for the production of the lentiviral supernatant. The shControl lentiviruses obtained were then used to infect HEK293 cells. The infected cells were seeded into culture plates 48 h post-infection and cultured in cell medium containing 1 μg/mL puromycin for screening stable shControl cells. The generation of the stable shControl cell line was confirmed by the co-expression of GFP.

### Coimmunoprecipitation to Confirm Substrate Ubiquitination in HEK293 Cells

The transfection of the plvx-UBE3A plasmid into shUBE3A and HEK293 cells was carried out according to Dharmacon transfection reagent’s protocol. After 48 h of transfection, cells were treated with 10 μM MG132 for 4 h. Cells were harvested and centrifuged to obtain cell lysates. Cell lysates were precleared, then incubated with anti-substrate antibodies at 4 °C for 2 h, followed by adding protein A/G PLUS agarose (Santa Cruz Biotechnology, sc-2003) and rocked gently overnight at 4 °C. The next day, the beads were washed four times with cold PBS, then resuspended in 1× SDS loading dye, and boiled. Immunoprecipitated samples were analyzed by western blotting that was probed with an anti-UB antibody to examine the ubiquitination level.

### Measurement of the Enzyme Activity of PDH

The PDH enzyme activity of HEK293 cells was measured using a colorimetric assay kit (K679) from Biovision (Milpitas, CA, USA), according to the manufacturer’s protocol.

### ECAR Measurement by a Seahorse XF Analyzer

Cells were seeded into 96-well microplates, with one well in each row or column left out for seeding. The empty wells were used as blank controls. The seeded plate was put into a 37 °C, 5% CO_2_ incubator to allow cells to adhere. The XF96 sensor cartridges were hydrated and placed in a 37 °C incubator, not supplemented with CO_2_ or oxygen/nitrogen. Before analysis, the medium of the microplate was replaced with a new medium consisting of the Seahorse XF Base Medium, 10 mM glucose, 2 mM sodium pyruvate, and 2 mM glutamine. Then, the microplate was placed in a CO_2_-free incubator for 1 h before the microplate was transferred to the analyzer. Measurements were conducted using final concentrations of 10 mM glucose, 2 μM oligomycin (OM), or 50 mM 2-deoxyglucose (2-DG).

### Animals and Diets

All experiments were performed in accordance with the NIH and Georgia State University guidelines for laboratory animals’ care and use and approved by the Committee for the Care and Use of Laboratory Animals in the Department of Biology, Georgia State University. Male C57BL/6J mice were purchased from the Jackson Laboratory (Bar Harbor, ME, USA). AAV control viruses were purchased from Addgene. AAV-UBE3A viruses were produced by the Penn Vector Core at the University of Pennsylvania. Mice were injected into the retro-orbital venous sinus with 10^11^ GC of AAV in a 50 μl volume. All animals were housed at 20–22 °C and 50 ± 10% humidity and fed a low fat, 10 kcal % fat diet (LFD, D12450B) or a high fat, 60 kcal % high-fat diet (HFD, D12492) obtained from Research Diets, Inc. (New Brunswick, NJ, USA).

### Sample Collection, Liver Protein, and Lipid Extraction

Mice were dissected, and livers were harvested immediately and snap frozen at −80 °C in liquid nitrogen before analysis. Blood was obtained by cardiac puncture and collected into EDTA-containing tubes, then centrifuged to get plasma. The liver for histology was fixed in 10% formalin at room temperature until further analysis. Livers were homogenized in RIPA buffer (25 mM Tris-HCl, pH 7.6, 150 mM NaCl, 1% NP-40, 1% sodium deoxycholate, 0.1% SDS, 1 mM PMSF) supplemented with a 1× protease cocktail inhibitor and a 1× phosphatase inhibitor and centrifuged to obtain liver proteins. A solution of CHCl_3_/MeOH (2/1, v/v) was used to extract lipids from the liver tissue. An aliquot of the organic phase of the extraction was collected, dried under nitrogen, and resuspended in 1% Triton X-100. Hepatic TG and cholesterol content and serum ketone were determined using commercially available kits.

### Hematoxylin and Eosin Staining

The formalin-fixed liver was embedded into paraffin and cut into 4 μm sections. Liver sections were stained with hematoxylin and eosin (H&E).

### Quantitative RT-PCR Analysis

The mRNA levels of lipogenic gene expression were assessed by quantitative RT-PCR. The total RNA was extracted from the liver samples using a Tri Reagent kit (Molecular Research Center, Cincinnati, OH). One-step RT-PCR analysis was conducted to measure the mRNA expression using an Applied Biosystems QuantStudio 3 (Thermo Fisher Scientific) with a TaqMan Universal PCR Master Mix kit (Thermo Fisher Scientific, Waltham, MA). The primer and probe pairs used in this analysis were purchased from Applied Biosystems (Thermo Fisher Scientific).

### Statistical Analysis

All statistical analyses were performed using GraphPad Prism 9.0.0 software, San Diego, CA, USA. All quantitative data were presented as mean ± SEM. Differences between the two groups were assessed by unpaired Student’s *t*-test. *p* < 0.05 was considered statistically significant.

## Results

### Identifying PDHA1 and ACAT1 as the Substrates of UBE3A by Orthogonal UB Transfer

To profile the substrates of UBE3A, we developed a method referred to as “orthogonal ubiquitin transfer” (OUT) that would enable the transfer of a UB mutant (*x*UB) with the R42E and R72E double mutations to the substrates of a specific E3, so their identities could be revealed by proteomics.^36,37^ OUT relies on engineered interactions of the *x*UB–*x*E1, *x*E1–*x*E2, and *x*E2–*x*E3 pairs for the assembly of the *x*E1–*x*E2–*x*E3 enzymatic cascade for the exclusive delivery of *x*UB to the ubiquitination targets of an E3 (“*x*” designates the engineered UB or the transfer enzymes that do not cross-react with their native partners in the UB transfer cascade). In a previous study, we constructed the OUT cascade with UBE3A/E6AP and used it to profile the substrate specificity of UBE3A in HEK293 cells. The OUT screen enabled us to assemble a substrate profile of UBE3A consisting of 130 potential substrates, and among them, we verified multiple new substrates of UBE3A in the cells, including kinases MAPK1, CDK1, and CDK4, protein arginine methyltransferase PRMT5, transcription factor β-catenin, UB-binding protein UbxD8, and O-linked *N*-acetyl glucosamine transferase (OGT).^37,38^ We further took advantage of the OUT cascade of UBE3A to compare its substrate profiles with and without the expression of HPV E6 and discovered that, besides p53, E6 stimulates UBE3A to ubiquitinate the importin α family of proteins, including KPNA1–3, to induce their degradation. This would enable HPV to suppress the nuclear transport of antiviral transcription factors and pivot the host defense against viral infection.^39^

To decipher the role of UBE3A in regulating cell metabolism, we found several enzymes involved in sugar and lipid metabolism in the substrate profiles of UBE3A, as revealed by the OUT screen (Supporting Information Table S1). For example, 6-phosphofructokinase PFKL and PFKP, phosphoacetylglucosamine mutase (PGM3), pyruvate dehydrogenase (PDH) E1 component subunit α (PDHA1), and galactokinase (GALK1) are enzymes participating in glycolysis and the TCA cycle. Acyl-CoA dehydrogenase ACAD9 and ACADSB, very-long-chain 3-oxoacyl-CoA reductase (HSD17B12), ACAT1, and acyl-coenzyme A thioesterase 9 (ACAT9) are enzymes contributing to fatty acid oxidation and biogenesis. In this study, we focused on PDHA1 and ACAT1 due to their importance in glucose and lipid metabolism. PDHA1 is a key component of the pyruvate dehydrogenase complex (PDC), which catalyzes pyruvate decarboxylation to produce acetyl-CoA and CO_2_, hence providing the primary link between glycolysis and the TCA cycle.^40−42^ PDHA1 downregulation has been indicated in various types of cancer cells to enhance glycolysis activity.^42,43^ Phosphorylation and acetylation of PDHA1 could inactivate the enzyme.^21,44^ ACAT1 catalyzes the condensation of two acetyl-CoAs to produce acetoacetyl-CoA as well as the reverse reaction that breaks down acetoacetyl-CoA into two acetyl-CoAs.^22,45^ In hepatic ketogenesis, ACAT1 favors acetoacetyl-CoA formation, whereas, in non-hepatic ketolysis, ACAT1 functions to break down acetoacetyl-CoA.^45^ Due to the essential roles of ACAT1 and PDHA1 in regulating glycolysis and lipid metabolism in the liver, we decided to verify the ubiquitination of PDHA1 and ACAT1 by UBE3A in HEK293 cells and study the physiological role of UBE3A in the regulation of its substrate protein stabilities and lipid metabolism in vivo.

### UBE3A Ubiquitinates PDHA1 and Regulates Its Stability in HEK293 Cells

**Figure 1:**
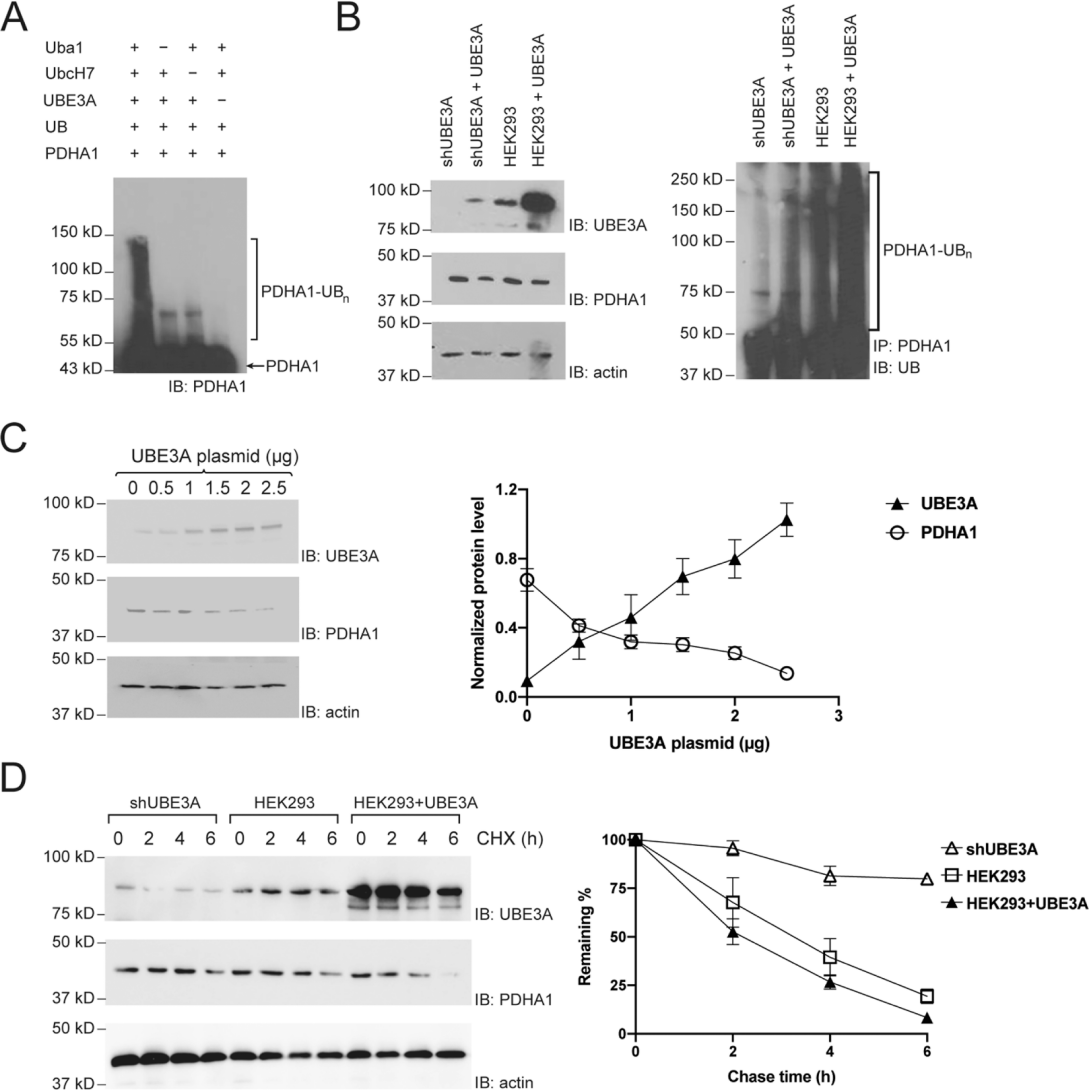
Verification of PDHA1 as a ubiquitination substrate of UBE3A. (A) UBE3A catalyzes PDHA1 ubiquitination in vitro. PDHA1 in vitro ubiquitination reactions were performed with the addition of Uba1, UbcH7, UBE3A, and UB. Control reactions were carried out with Uba1, UbcH7, or UBE3A excluded from the assays. Reactions were quenched by boiling in SDS-PAGE loading buffer and analyzed by western blotting probed with an anti-PDHA1 antibody. (B) UBE3A induces PDHA1 ubiquitination in HEK293 cells. Before harvesting, cells were treated with 10 μM MG132 for 4 h. The left panels show protein expression in the cell lysate of four different cell populations as probed by various antibodies. The right panel shows that UBE3A markedly increased PDHA1 ubiquitination in HEK293 cells, as demonstrated by immunoprecipitation of PDHA1 proteins, followed by western blotting analysis with an anti-UB antibody. (C) UBE3A downregulates PDHA1 steady-state levels in HEK293 cells. Cells were transiently transfected with an increasing amount of the plvx-UBE3A plasmid. PDHA1 protein levels were assayed with an anti-PDHA1 antibody. Quantitative analysis of PDHA1 protein levels in line with UBE3A expression was shown in the right panel. (D) UBE3A accelerates PDHA1 degradation in HEK293 cells. CHX chase assays were carried out with shUBE3A, blank HEK293 cells, and cells overexpressing UBE3A. The cells were treated with 100 μg/mL CHX for 0, 2, 4, and 6 h. Quantitative analysis of the PDHA1 protein level is shown in the right panel. Data points show mean ± S.E. of three experiments. The vertical bars in (C,D) represent SEM from three independent experiments (*n* = 3).

We first performed an in vitro ubiquitination assay to verify the ubiquitination of PDHA1 by UBE3A with both the substrate protein and the E3 ligase enzyme expressed from *E. coli*. In the reconstituted reactions, Uba1 (E1), UbcH7 (E2), and UBE3A (E3) were combined with PDHA1, and wild-type UB and ATP were added to initiate the reaction. Control reactions were set up in parallel, excluding the addition of Uba1, UbcH7, or UBE3A, to verify the dependence of substrate ubiquitination on the UB transfer cascades. We found UBE3A catalyzed the ubiquitination of PDHA1 in the presence of Uba1 and UbcH7, as evidenced by the formation of the higher molecular-weight forms of PDHA1 detected on the western blot with an anti-PDHA1 antibody (Figure 1A). When any cascade proteins were omitted, the ubiquitinated form of PDHA1 did not form (Figure 1A), suggesting that the UB transfer through UBE3A is responsible for PDHA1 ubiquitination in vitro. UBE3A relies on C820 as the catalytic Cys residue in the HECT domain for the formation of a thioester conjugate with UB and the transfer of UB to the substrate proteins.^46^ We generated the C820A mutant of UBE3A and found that it could not catalyze the ubiquitination of PDHA1 (Figure S1A).

We then examined PDHA1 ubiquitination levels in HEK293 cells with knockdown or overexpression of UBE3A and with MG132 treatment of the cells to inhibit protein degradation by the proteasome. We screened the efficiency of shUBE3A plasmids in gene silencing and used the plasmid of the highest efficiency to establish stable cells with a silenced expression of UBE3A (shUBE3A) (Figure S1B).^37^ We overexpressed UBE3A in shUBE3A cells and HEK293 cells, immunoprecipitated PDHA1, and probed its ubiquitination levels with an anti-UB antibody. We also examined PDHA1 ubiquitination levels in shUBE3A cells and HEK293 cells with endogenous UBE3A expression. We found that PDHA1 ubiquitination was largely undetectable in shUBE3A cells with a silenced expression of the E3 (Figure 1B). In contrast, re-expressing UBE3A in shUBE3A cells partially recovered PDHA1 ubiquitination, while over-expressing UBE3A in HEK293 cells further enhanced PDHA1 ubiquitination (Figure 1B). We also assayed the ubiquitination of PDHA1 in HEK293 cells stably transfected with an shControl plasmid. We found that there was comparable background ubiquitination of PDHA1 in HEK293 cells and shControl cells, while the transfection of the expression plasmid of UBE3A significantly enhanced PDHA1 ubiquitination (Figure S1C). These results verified that UBE3A would recognize PDHA1 as a ubiquitination target in HEK293 cells.

To determine if UBE3A regulates the degradation of PDHA1 by ubiquitination, we transiently transfected UBE3A expression plasmids with titration into HEK293 cells and measured the steady-state levels of PDHA1 after 24 h of transfection. We found that UBE3A over-expression decreased PDHA1 protein levels in a dose-responsive manner (Figure 1C). We also carried out a cycloheximide (CHX) pulse-chase assay to investigate whether UBE3A accelerated the degradation of PDHA1. We treated the cells of shUBE3A, HEK293, and HEK293 with UBE3A overexpression with CHX that inhibited protein synthesis and probed the PDHA1 levels by western blot after different durations of exposure to CHX. We found that PDHA1 protein was degraded more rapidly in UBE3A-overexpressed cells than in control HEK293 cells, while the protein remained largely unchanged in shUBE3A cells along the time course (Figure 1D). These results indicated that UBE3A-mediated ubiquitination would induce the degradation of PDHA1 in HEK293 cells.

### UBE3A-Induced PDHA1 Degradation Enhances the Glycolytic Activity in HEK293 Cells

**Figure 2:**
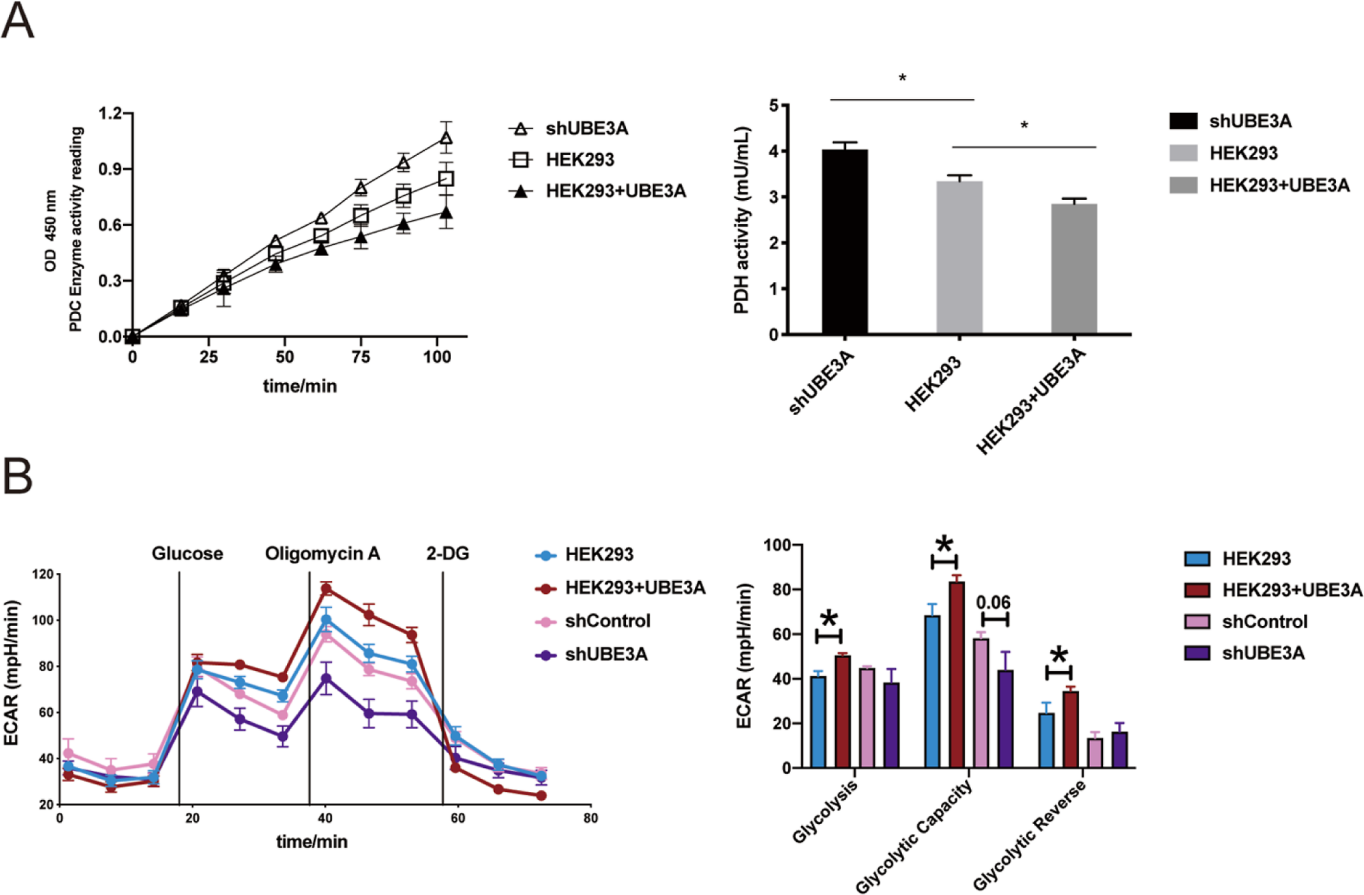
Enhancement of glycolysis activities in the cell as a result of PDHA1 degradation triggered by UBE3A. (A) UBE3A decreases PDH enzyme activity in HEK293 cells. Activities of the PDH enzyme in shUBE3A, blank HEK293, and cells overexpressing UBE3A were measured using a colorimetric kit from Biovision. In the assay, PDH converts pyruvate into an intermediate with the coproduction of NADH, which reduces the developer to a colored product with strong absorbance at 450 nm. Cells (1 × 10^6^) were homogenized in 100 μL ice-cold PDH assay buffer. 5–50 μL of the sample was added to each well, and the volume in the well was adjusted to 50 μL with the PDH assay buffer. Standard samples of NADH were added into a series of wells in the 96-well plate. A reaction mixture containing PDH assay buffer, a PDH developer, and a PDH substrate was added to each well. The absorbance of 450 nm was measured in the kinetic mode for 100 min. PDH activity was calculated according to the NADH standard curve and plotted in the right panel. (B) UBE3A enhances glycolytic activity in HEK293 cells. ECAR (extracellular acidity rate) was measured using a Seahorse XFe96 Analyzer. Cells were inoculated into the XFe96 cell culture microplate at a density of 1 × 10^4^ cells/well and then cultured overnight. Before analysis, the medium of the microplate was replaced with a new medium consisting of the Seahorse XF Base Medium, 10 mM glucose, 2 mM sodium pyruvate, and 2 mM glutamine. The microplate was placed in a CO_2_-free incubator for 1 h before it was transferred to the analyzer. Measurements were conducted using final concentrations of 10 mM glucose, 2 μM OM, or 50 mM 2-deoxyglucose (2-DG). Glycolysis and glycolytic capacity were calculated and plotted in the right panel.

PDHA1 is a key catalytic unit of PDC that directs the flux of the pyruvate to the TCA cycle in the form of acetyl CoA instead of to the path of lactate conversion to enable glycolysis under anaerobic conditions. Since PDHA1 is subject to UBE3A-mediated proteasomal degradation (Figure 1), we determined the biological outcome of the loss of PDHA1 due to UBE3A-catalyzed ubiquitination by assessing the glycolytic activity in HEK293 cells. We measured pyruvate dehydrogenase (PDH) activity in shUBE3A cells, HEK293 cells, and HEK293 cells with UBE3A overexpression using a colorimetric kit.^47^ Consistent with its role in decreasing PDHA1 protein stability, UBE3A overexpression in the cells decreased PDH activity by 20% compared to HEK293 cells. On the other hand, there was a 20% increase in PDH activity in shUBE3A cells compared to HEK293 cells, suggesting that the silenced UBE3A expression would lead to a stabilization of PDHA1 and enhancement of its enzymatic activity in the cells (Figure 2A).

Since PDH inactivation is associated with elevated aerobic glucose utilization,^48^ we assessed glycolytic capacities in shUBE3A, HEK293, and HEK293 cells with UBE3A-overexpression using the Seahorse Extracellular Flux Analyzer.^49^ Thereby, we can perform the real-time measurement of the glycolytic capacity and glycolytic reserve in the cells with different levels of UBE3A expression by sequential injection of glucose, OM, and 2-DG (2-deoxy-d-glucose) using the extracellular acidification rate (ECAR) as a parameter for proton release by glycolysis. ECAR values were expressed in units of mpH min^–1^, which follow the changes in pH in the media surrounding the cells as a result of acidification, mainly due to glycolytic proton efflux. As expected, we observed that cells with UBE3A overexpression showed significantly higher basal glycolysis, glycolytic capacity, and glycolytic reserve than the mock HEK293 cells (Figure 2B), suggesting that increased UBE3A expression would lead to enhanced glycolysis by inducing the degradation of PDHA1 and downregulating its enzymatic activity in HEK293 cells. As a control, cells stably transfected with the shControl plasmid showed similar glycolysis activity as the parent HEK293 cells. The different expression levels of UBE3A in various types of cells used in the assay were confirmed by western blotting (Figure S1D).

### UBE3A Ubiquitinates ACAT1 and Regulates Its Stability in HEK293 Cells

**Figure 3:**
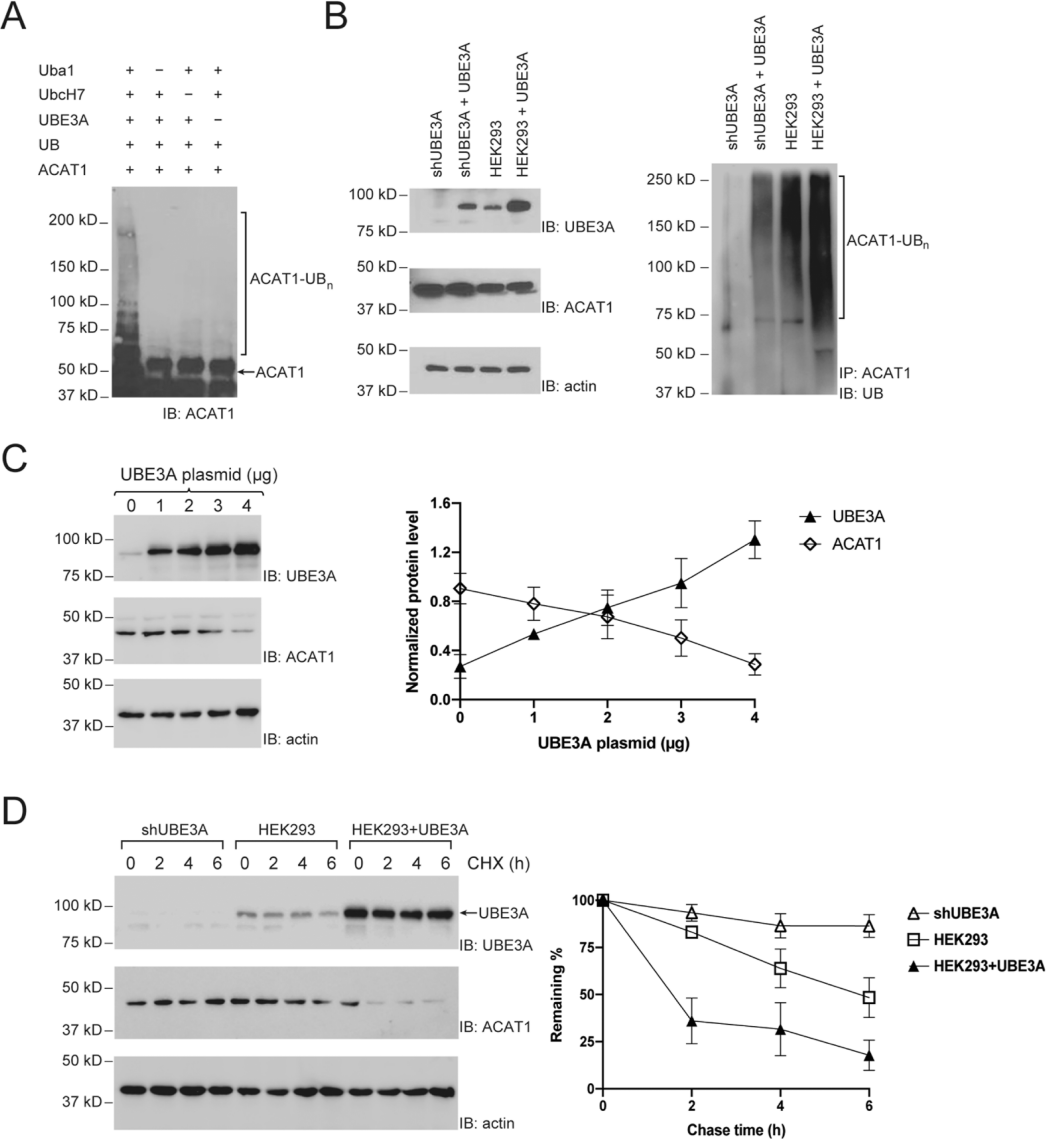
Verification of ACAT1 as a ubiquitination substrate of UBE3A. (A) UBE3A catalyzes ACAT1 ubiquitination in vitro. ACAT1 in vitro ubiquitination reactions were performed with the addition of Uba1, UbcH7, UBE3A, and UB. Control reactions were carried out with Uba1, UbcH7, or UBE3A excluded from the assays. Reactions were quenched by boiling in SDS-PAGE loading buffer and analyzed by western blotting probed with an anti-ACAT1 antibody. (B) UBE3A induces ACAT1 ubiquitination in HEK293 cells. Before harvesting, cells were treated with 10 μM MG132 for 4 h. The left panels show expression levels of UBE3A and ACAT1 in various cell populations, as probed by specific antibodies. The right panel shows that the expression of UBE3A markedly increased ACAT1 ubiquitination in HEK293 cells, as demonstrated by the immunoprecipitation of ACAT1 proteins, followed by western blotting analysis with an anti-UB antibody. (C) UBE3A downregulates ACAT1 steady-state levels in HEK293 cells. Cells were transiently transfected with an increasing amount of the plvx-UBE3A plasmid. ACAT1 protein levels were assayed with an anti-ACAT1 antibody. Quantitative analysis of ACAT1 protein level in line with the UBE3A expression was shown in the right panel. (D) UBE3A accelerates ACAT1 protein degradation in HEK293 cells. A CHX chase assay was carried out with shUBE3A cells, blank HEK293 cells, and cells overexpressing UBE3A. The cells were treated with 100 μg/mL CHX for 0, 2, 4, and 6 h. Quantitative analysis of the protein level was shown in the right panel. Data points show mean ± S.E. of three experiments. The vertical bars in (C,D) represent SEM from three independent experiments (*n* = 3).

Following a similar assaying scheme, we verified ACAT1 ubiquitination in vitro by UBE3A and found that the ubiquitination reaction was dependent on Uba1, UbcH7, and UBE3A for transferring UB to ACAT1 (Figure 3A). Furthermore, the catalytically inactive C820A mutant of UBE3A lost its capacity to ubiquitinate ACAT1 in the reconstituted reaction (Figure S2A). We also assessed the ubiquitination of ACAT1 in HEK293 cells and cells with UBE3A overexpression after treating the cells with the proteasome inhibitor MG132 to accumulate ubiquitinated species. The ubiquitination assays, which were conducted by the immunoprecipitation of ACAT1, followed by immunoblotting with the anti-UB antibody, showed that UBE3A deficiency diminished the ACAT1 ubiquitination in shUBE3A cells, which was substantially recovered by UBE3A re-expression (Figure 3B). Similarly, enhanced ubiquitination of ACAT1 was observed in UBE3A-overexpressed cells compared to the control HEK293 cells (Figure 3B). We also found that UBE3A overexpression enhanced the ubiquitination of ACAT1 in shControl cells (Figure S2B). These results confirmed that ACAT1 is a substrate of UBE3A for ubiquitination.

To determine if UBE3A regulates the stability of ACAT1 in HEK293 cells, we transfected the HEK293 cells with various amounts of UBE3A expression plasmids. We found that UBE3A overexpression dose-dependently decreased ACAT1 protein levels (Figure 3C). The CHX chase assay also showed that the over-expression of UBE3A in HEK293 cells accelerated the degradation of ACAT1 compared to the control HEK293 cells, while the silenced UBE3A expression in shUBE3A cells stabilized the ACAT1 protein in the cells (Figure 3D). These results established a UBE3A–ACAT1 regulatory axis that would control ACAT1 protein degradation in the cells through the ubiquitination reaction.

### UBE3A Over-Expression Promotes Hepatic Steatosis by Regulating the ACAT1 Protein

**Figure 4:**
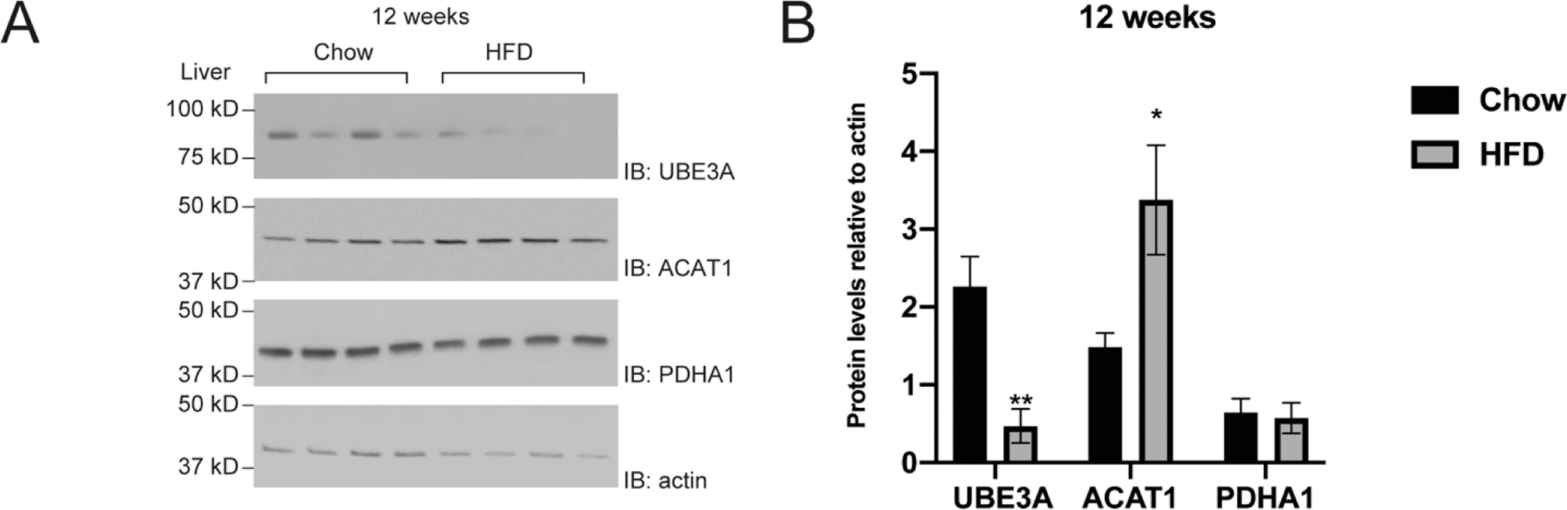
Change of the protein levels of UBE3A, ACAT1, and PDHA1 in the livers of mice fed with a high-fat diet. (A) High-fat diet (HFD) reduces UBE3A protein levels and enhances ACAT1 protein levels. 8 week old male C57BL/6J mice were fed the chow diet or HFD for 12 weeks. Liver tissue was homogenized to extract the lysates. Proteins were analyzed by western blotting with anti-UBE3A, anti-ACAT1, and anti-PDHA1 antibodies. (B) Quantitative analysis of the protein levels shown in (A). Data points show mean ± S.E. of three or more experiments. *p < 0.05 vs chow diet. **p < 0.01 vs chow diet (*n* = 4 mice per group).

**Figure 5:**
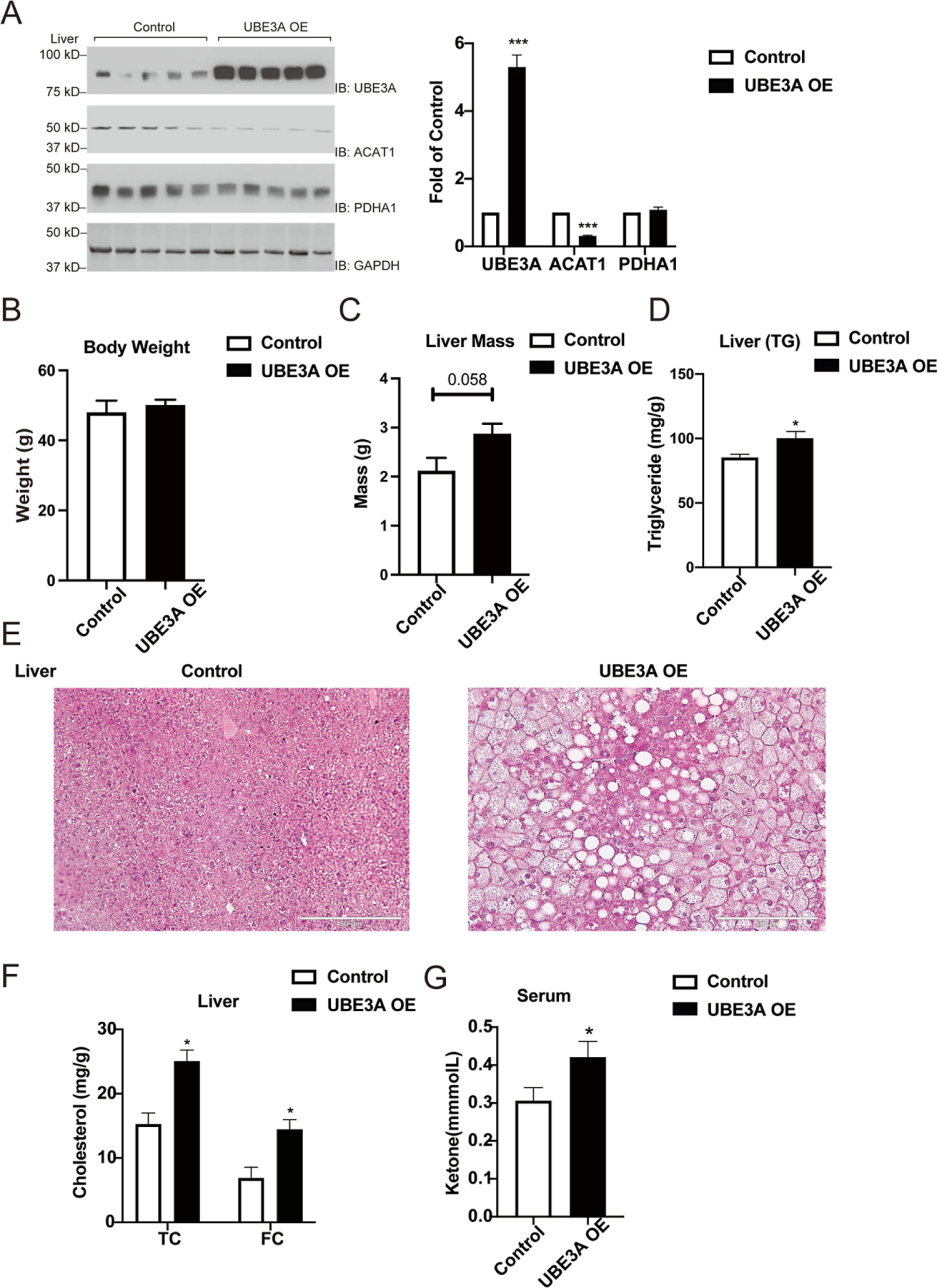
Effect of the UBE3A overexpression in the mouse liver on ACAT1 levels and the development of hepatic steatosis. (A) UBE3A overexpression reduces ACAT1 protein levels in the liver. Liver proteins were analyzed by western blotting and probed with specific antibodies. Quantitative analysis of protein levels was plotted in the right panel. (B) Body weight of mice infected with the AAV UBE3A expression virus or the control mice infected with the AAV GFP virus. (C) UBE3A overexpression increases liver weight. (D) UBE3A overexpression promotes liver TG contents. Liver TG contents were measured using a colorimetric enzymatic assay. (E) UBE3A overexpression promotes hepatic steatosis. Liver samples were taken from all the mice and fixed with 10% formaldehyde in PBS for 24 h. They were washed with tap water, dehydrated in alcohol, and embedded in paraffin. Sections 3–5 μm thick were mounted in glass slides covered with silane. Hematoxylin and eosin (H&E) stains were performed on each slide. (F) UBE3A overexpression increases TC and FC in the liver. Liver cholesterol contents were measured using an enzymatic assay. (G) UBE3A overexpression increases serum ketone contents. Serum ketones were measured using an enzymatic assay. Data points show mean ± S.E. of three or more experiments. *p < 0.05 versus WT mice. **p < 0.01 versus WT mice. ***p < 0.001 versus WT mice (*n* = 5 mice per group).

To determine the physiological significance of UBE3A and its substrates in hepatic metabolism, we measured the protein levels of UBE3A, PDHA1, and ACAT1 in the liver of mice fed with a chow diet or HFD for 12 weeks. We observed that HFD feeding attenuated UBE3A protein levels in the liver, which was associated with enhanced ACAT1 protein levels compared to chow diet feeding. However, there was no difference in the liver PDHA1 protein between chow-fed and HFD-fed mice (Figure 4). We reasoned that the forced UBE3A expression in the liver might rectify HFD-induced over-expression of ACAT1 protein, which may contribute to hepatic dysfunction in lipid metabolism. We therefore injected AAV virus expressing UBE3A into 8-week old C57BL/6J mice retro-orbitally for liver-targeted Ube3a gene transfer and fed these mice with HFD for 10 weeks. Consistent with our findings in the in vitro experiments, the overexpression of UBE3A led to a marked downregulation of ACAT1 protein contents (Figure 5A). Although there was no change in body weight (Figure 5B), we observed increases in liver mass and liver triglyceride (TG) contents in AAV Ube3a virus-infected mice compared to the control mice infected with the AAV GFP virus (Figure 5C,D). In consistency, H&E staining also revealed a noticeable augmentation in lipid accumulation in the liver with UBE3A overexpression (Figure 5E). To uncover the pathways responsible for the enhanced lipid deposition in the UBE3A-overexpressing liver, we measured the expression of genes involved in lipid metabolism including lipogenesis and TG synthesis. Quantitative RT-PCR analysis revealed no change in the expression of lipogenic genes including Srebp1c, Acc2, Fasn, Scd1, and TG-synthesizing genes Dgat1 and Dgat2 in the Ube3a-overexpressing liver compared to the control (Figure S3). These data suggest that pathways other than lipogenesis might be responsible for the enhanced TG accumulation observed in the UBE3A-overexpressing liver.

Since the liver is the major cholesterol biosynthetic organ, we measured total cholesterol (TC) and free cholesterol (FC) in the liver tissue. We found that mice infected with the AAV Ube3a virus exhibited higher TC and FC levels than the control mice infected with the AAV GFP virus (Figure 5F). Because the liver is responsible for generating ketone bodies and ACAT1 is associated with ketone metabolism,^50^ we examined the level of serum ketone bodies and found that the mice with UBE3A overexpression displayed elevated serum ketone levels in comparison to the control mice (Figure 5G). This suggests that a decreased ACAT1 activity for breaking down acetoacetyl CoA in mice is associated with the overexpression of UBE3A that would induce the ubiquitination and degradation of ACAT1. Collectively, these results indicated that UBE3A overexpression in the mouse liver gave rise to enhanced fat deposition as well as serum ketone accumulation, which is associated with the downregulation of ACAT1.

## Discussion

NAFLD is a serious metabolic disorder that stems from the dysregulation of hepatic lipid metabolism. The central feature of NAFLD is hepatic steatosis, the deposition of excess TG in the liver.^51^ Excess hepatic FFAs, due to a combination of increased FFA influx from dietary sources and adipose lipolysis, would enhance de novo lipogenesis and decrease FFA utilization via hepatic lipid oxidation and secretion and play a key role in the initiation and development of hepatic steatosis.^51^ While much effort has been devoted to studying the transcriptional regulation of metabolic pathways during the development of hepatic steatosis, little is known about the post-translational modification of metabolic enzymes in this process, especially through ubiquitination-mediated mechanisms. Therefore, we employed a unique approach based on the OUT platform to identify the metabolic enzymes as ubiquitination targets of UBE3A, which has been shown to prevent HFD-induced hepatic steatosis in mice.^35^ We further verified the ubiquitination of PDHA1 and ACAT1 by UBE3A in HEK293 cells and studied the physiological role of UBE3A in regulating the stabilities of its substrate proteins and lipid metabolism in vivo. Our data indicate that the down-regulation of UBE3A by nutrient-rich diets may result in less ubiquitination of metabolic enzymes such as ACAT1 and subsequent up-regulation of ACAT1 protein stability, thereby promoting hepatic lipid accumulation.

ACAT1 is a mitochondrial enzyme that reversibly catalyzes the formation of acetoacetyl-CoA with two molecules of acetyl-CoA during ketone biosynthesis or breakdown.^45^ We confirmed that UBE3A targets ACAT1 for ubiquitination in HEK293 cells, and the overexpression of UBE3A accelerates the degradation of ACAT1 in the cell (Figure 3). Matching with the regulatory relationships between UBE3A and ACAT1 that we identified in the cell, we found that HFD feeding reduced the UBE3A level in the mouse liver with a parallel increase of the ACAT1 level (Figure 4). We also probed for ACAT1 in the mouse liver with and without the UBE3A overexpression and found that enhanced UBE3A expression led to a substantial downregulation of ACAT1, confirming the role of UBE3A in destabilizing ACAT1 through ubiquitination (Figure 5A). Furthermore, mice with enhanced UBE3A expression manifested hepatic steatosis symptoms, such as an elevated deposition of TG, lipid, and cholesterol in the liver and higher ketone levels in the serum (Figure 5D–G). These effects may result from suppressed ACAT1 activity by UBE3A that would tip the balance of lipid metabolism toward keto and lipid genesis. Other mechanisms may also increase serum ketone levels with decreased activity of ACAT1. For instance, apart from its thiolase activity in ketone metabolism, ACAT1 has been shown to act as a lysine acetyltransferase that acetylates PDHA1,^22^ a key component of PDC that breaks pyruvates into acetyl-CoAs through decarboxylation. The acetylation of PDHA1, however, inhibits PDC, resulting in the decreased production of acetyl-CoA and increased accumulation of pyruvate-converted lactates. It is conceivable that reduced ACAT1 activity by UBE3A overexpression may maintain an active state of PDC due to less acetylation of PDHA1. The accumulation of acetyl-CoA produced by active PDC may therefore provide ample building blocks for ketone body formation, which eventually contributes to the increased ketone contents in circulation, as we observed.

We also found that UBE3A recognizes PDHA1 as a substrate protein, and the degradation of PDHA1 mediated by UBE3A enhances glycolysis in HEK293 cells. Since the PDH complex directs the flux of pyruvate to enter the TCA cycle and low PDHA1 activity would suppress the TCA cycle and enhance glycolysis, we identified a role of UBE3A in regulating the balance between glycolysis and the TCA cycle by targeting PDHA1 for ubiquitination and degradation. We found that PDHA1 has similar expression levels in the mouse liver with or without the overexpression of UBE3A, suggesting that UBE3A-induced degradation may not play an essential role in regulating PDHA1 stability in the mouse liver (Figures 4A and 5A). Other factors may cooperate with UBE3A to affect the PDHA1 level and activity in the liver cell. It is known that PGC1α, a transcription coactivator regulating mitochondrial metabolism, enhances the expression of PDHA1.^52^ Also, the activity of PDHA1 is regulated by phosphorylation and acetylation, and these post-translational modifications may affect the ubiquitination and stability of PDHA1 in the liver tissue.^20,21^ It was reported that the E1β subunit of PDH (PDHB) that forms a heterotetrameric complex with PDHA1 is ubiquitinated and degraded by the proteasome upon its phosphorylation by the protein tyrosine kinase activity of the epidermal growth factor receptor.^53^ The E3 ligase responsible for the ubiquitination of PDHB is not known. Future work is warranted to assay if UBE3A also targets PDHB for ubiquitination and if glycolysis would be regulated by UBE3A expression in the mouse liver.

Our work suggests a ubiquitination-mediated regulatory axis between UBE3A and key metabolic enzymes PDHA1 and ACAT1 with which the liver cells can properly regulate glycolysis, ketogenesis, and lipid synthesis in response to the diet change that may promote NAFLD. As mentioned before, deletion or inactivation of the maternally inherited *UBE3A* gene results in the absence of the functional UBE3A protein in the brain, causing Angelman syndrome,^54,55^ which is a neurodevelopmental disorder characterized by delayed development, lack of speech, intellectual disability, seizure, and other symptoms.^56,57^ It was also reported that obesity is common with Angelman syndrome.^58,59^ The cohabitation of obesity with Angelman syndrome suggests that a loss-of-function mutation in UBE3A potentially causes dysregulation of metabolic pathways and energy metabolism. Indeed, *Ube3a*^*+/–*^ mice are prone to HFD-induced obesity and fatty liver development, suggesting an antagonistic role of UBE3A in obesity-related pathogenesis.^35^ Mechanistically, a recent report from Kim et al. demonstrated that UBE3A suppresses lipogenic gene expression by targeting the histone methyltransferase MLL4 for ubiquitination and degradation, resulting in the inhibition of hepatic steatosis.^35^ We found that HFD feeding down-regulates the UBE3A level in the mouse liver, suggesting the sensitivity of UBE3A to over nutritional conditions (Figure 4). Furthermore, we found that the forced expression of UBE3A in the mouse liver promotes hepatic steatosis as manifested by increased lipid deposition in the liver and higher levels of liver cholesterol and serum ketones (Figure 5D–G). These results are different from Kim’s findings. The exact reason for this discrepancy is not clear, but the two studies employed different models in which distinct genetic approaches were used to engineer the UBE3A expression in the liver. Kim’s paper utilized genetic models with a gain or loss of UBE3A globally (UBE3A knockout or Ube3a-Tg), in which the liver phenotypes may be confounded by the effects of UBE3A in other tissues, where UBE3A deletion or overexpression takes place. On the other hand, our study utilized AAV-mediated UBE3A overexpression in the liver, where cells other than hepatocytes presumably overexpress UBE3A due to an indiscriminate AAV infection. Cross-talk between hepatocytes and non-parenchymal cells may have an impact on hepatic lipid metabolism. Our study and Kim’s study shed light on the complex role of UBE3A in regulating the response of metabolic pathways to nutritional cues. Nonetheless, further studies are warranted to explore this discrepancy and elucidate how UBE3A regulates liver lipid metabolism.

The cross-regulatory mechanism between protein ubiquitination and metabolic pathways revealed in this work may widely exist in the cell to provide checkpoints for the precise control of metabolic flux. So far, a few examples have been reported on the ubiquitination of metabolic enzymes to regulate their activities in the cell.^60^ E3 ligase TRAF6 was shown to ubiquitinate hexokinase-2 (HK-2), the first enzyme in the glycolysis pathway.^61^ The ubiquitination of HK-2 leads to its recognition by autophagy receptors for the degradation and inhibition of glycolysis. 6-phosphofructocto-2-kinase/fructose-2,6-bisphosphotatse isoform 3 (PFKB3) is another enzyme in the glycolysis pathway, and its ubiquitination by the E3 ligase APC-Cdh1 in the neuronal cells results in the degradation of the enzyme and suppression of glycolysis. This would favor pentose phosphate pathways for glucose metabolism to generate reduced glutathione.^62^ In another example, CHIP E3 was found to ubiquitinate pyruvate kinase M2 (PKM2), a glycolytic enzyme and a critical regulator of glycolysis in tumors. PKM2 ubiquitination by CHIP would destabilize PKM2 and inhibit tumor growth by suppressing the Warburg effect.^63^ Here, we were guided by the substrate profile of UBE3A generated by the OUT screen and authenticated ACAT1 and PDHA1 as ubiquitination targets of UBE3A and established a role of the E3 in regulating glycolysis and lipid metabolism. We validated OUT as an empowering discovery platform for mapping cross-regulations between ubiquitination and metabolism pathways in the cell. We expect that the substrate profiles of other E3s generated by OUT, such as CHIP, E4B, and Rsp5, may also be utilized to reveal the mechanisms for maintaining a healthy cell metabolism and study how their dysregulation could be causative for metabolic diseases.^64,65^

## Conclusions

In sum, we here identified PDHA1 and ACAT1 as ubiquitination targets of the E3 ligase UBE3A. Our data indicate that UBE3A-mediated ubiquitination accelerates the proteasomal degradation of PDHA1 and reduces their enzymatic activities in the cells. UBE3A regulation of ACAT1 protein stability also bears physiological significance, as overexpressing UBE3A in the mouse liver downregulates ACAT1 protein contents, increases circulating ketone levels, and promotes hepatic steatosis. Our findings demonstrate a potential role for UBE3A and its target proteins in regulating hepatic nutrient metabolism.

## Supporting Information Available

Ubiquitination of PDHA1 and ACAT1, and mRNA levels of lipogenic genes in the UBE3A-overexpressing liver (PDF)
UBE3A substrates relevant to metabolic pathways (XLSX)

## Accession Codes

PDHA1: P08559 (UniProt); ACAT1: P24752 (UniProt); UB: P62975 (UniProt); Uba1: P22314 (UniProt); UbcH7: P68036 (UniProt); UBE3A: Q05086 (UniProt).

## Abbreviations

ACAT1: acetyl-CoA acetyltransferase 1
CHX: cycloheximide
ECAR: extracellular acidification rate
FC: free cholesterol
FFA: free fatty acids
HFD: high-fat diet
NAFLD: nonalcoholic fatty liver disease
NASH: nonalcoholic steatohepatitis
OUT: orthogonal ubiquitin transfer
PDC: pyruvate dehydrogenase complex
PDHA1: pyruvate dehydrogenase A1
TC: total cholesterol
TCA: tricarboxylic acid
T2DM: type-2 diabetes mellitus
UB: ubiquitin
E1: UB-activating enzyme
E2: UB-conjugating enzyme
E3: UB ligases
