# Supplementary material for: Effects of UBE3A on Cell and Liver Metabolism through the Ubiquitination of PDHA1 and ACAT1
Source: Biochemistry. 2023 Mar 15;62(7):1274–86. doi: 10.1021/acs.biochem.2c00624 (PMC10077595; doi:10.1021/acs.biochem.2c00624)
Supplement: Supplementary file 1 — bi2c00624_si_001.pdf [file bi2c00624_si_001.pdf]

Supporting Information for

Effects of UBE3A on Cell and Liver Metabolism Through the Ubiquitination of PDHA1 and ACAT1

Kangli Peng [1,2,‡], Shirong Wang[3, ‡], Ruochuan Liu,[2] Li Zhou,[2] Geon H. Jeong,[2] In Ho Jeong,[2] Xianpeng Liu,[4] Hiroaki Kiyokawa,[4] Bingzhong Xue[3], Bo Zhao[1, *], Hang Shi[3, *] and Jun Yin[2, *]

[1]Engineering Research Center of Cell and Therapeutic Antibody, Ministry of Education, and School of Pharmacy, Shanghai Jiao Tong University, Shanghai 200240, China.

[2]Department of Chemistry, Center for Diagnostics and Therapeutics, Georgia State University, Atlanta, GA 30303, USA

[3]Department of Biology, Georgia State University, Atlanta, GA 30303, USA

[4]Department of Pharmacology, Northwestern University, Chicago, IL 60611, USA

‡ These authors contributed equally to this work.

*Corresponding authors.

Corresponding authors:

Bo Zhao- Engineering Research Center of Cell and Therapeutic Antibody, Ministry of Education, and School of Pharmacy, Shanghai Jiao Tong University, Shanghai, China. Email: bozhao@sjtu.edu.cn

Hang Shi- Department of Biology, Georgia State University, Atlanta, GA 30303, USA. Email: hshi3@gsu.edu

Jun Yin- Department of Chemistry, Center for Diagnostics and Therapeutics, Georgia State University, Atlanta, GA 30303, USA. Email: junyin@gsu.edu

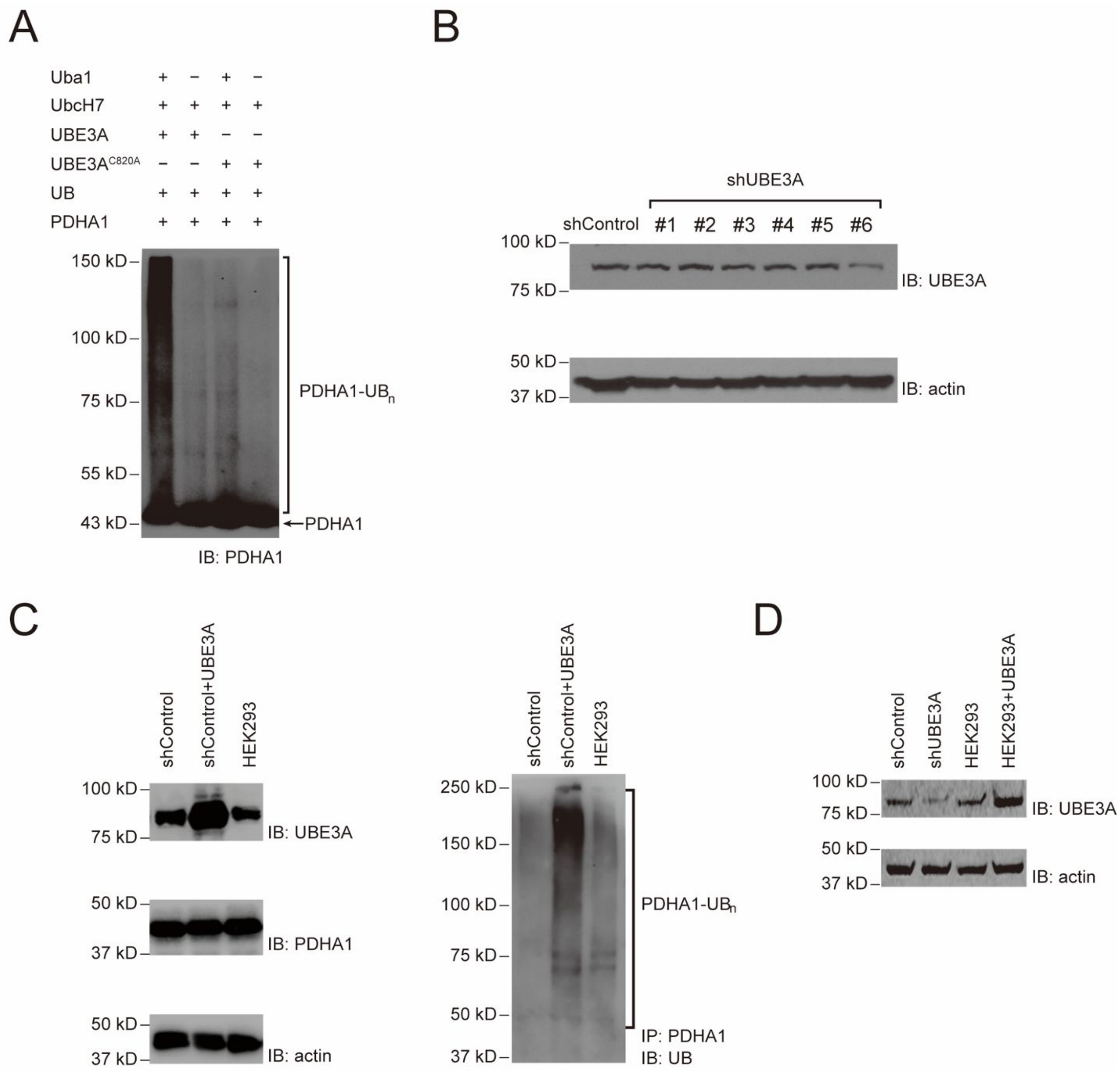

Figure S1. Verification of PDHA1 as a ubiquitination substrate of UBE3A with the C820A mutant of UBE3A and the shControl cells. (A) Reconstituted ubiquitination of PDHA1 with wild-type UBE3A and its C820A mutant that is catalytically inactive in UB transfer. (B) Screening the activity of shUBE3A plasmids in silencing the expression of UBE3A in HEK293 cells. ShUBE3A plasmids from Horizon Discovery (#1-6) were individually transfected into HEK293 cells, and the expression level of UBE3A in the cell was probed on the western blot with an anti-UBE3A antibody. (C) The expression of UBE3A in shControl cells enhanced the ubiquitination of PDHA1. PDHA1 was immunoprecipitated from shControl cells, shControl cells overexpressing UBE3A, and HEK293 cells, and its ubiquitination level was analyzed by western blot with an anti-UB antibody. (D) The different expression levels of UBE3A in various types of cells used in the Seahorse assay.

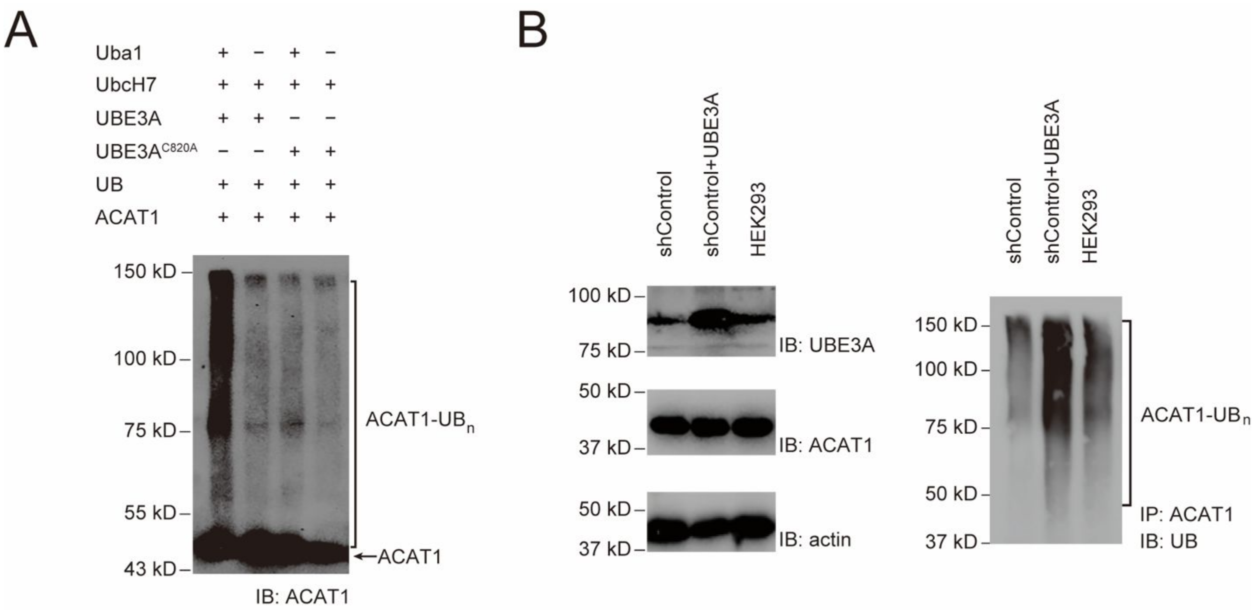

Figure S2. Verification of ACAT1 as a ubiquitination substrate of UBE3A with the C820A mutant of UBE3A and the shControl cells. (A) Reconstituted ubiquitination of ACAT1 with wild-type UBE3A and the C820A mutant of UBE3A. (B) The expression of UBE3A in shControl cells enhanced the ubiquitination of ACAT1. ACAT1 was immunoprecipitated from shControl cells, shControl cells overexpressing UBE3A, and HEK293 cells, and its ubiquitination level was analyzed by western blot with an anti-UB antibody.

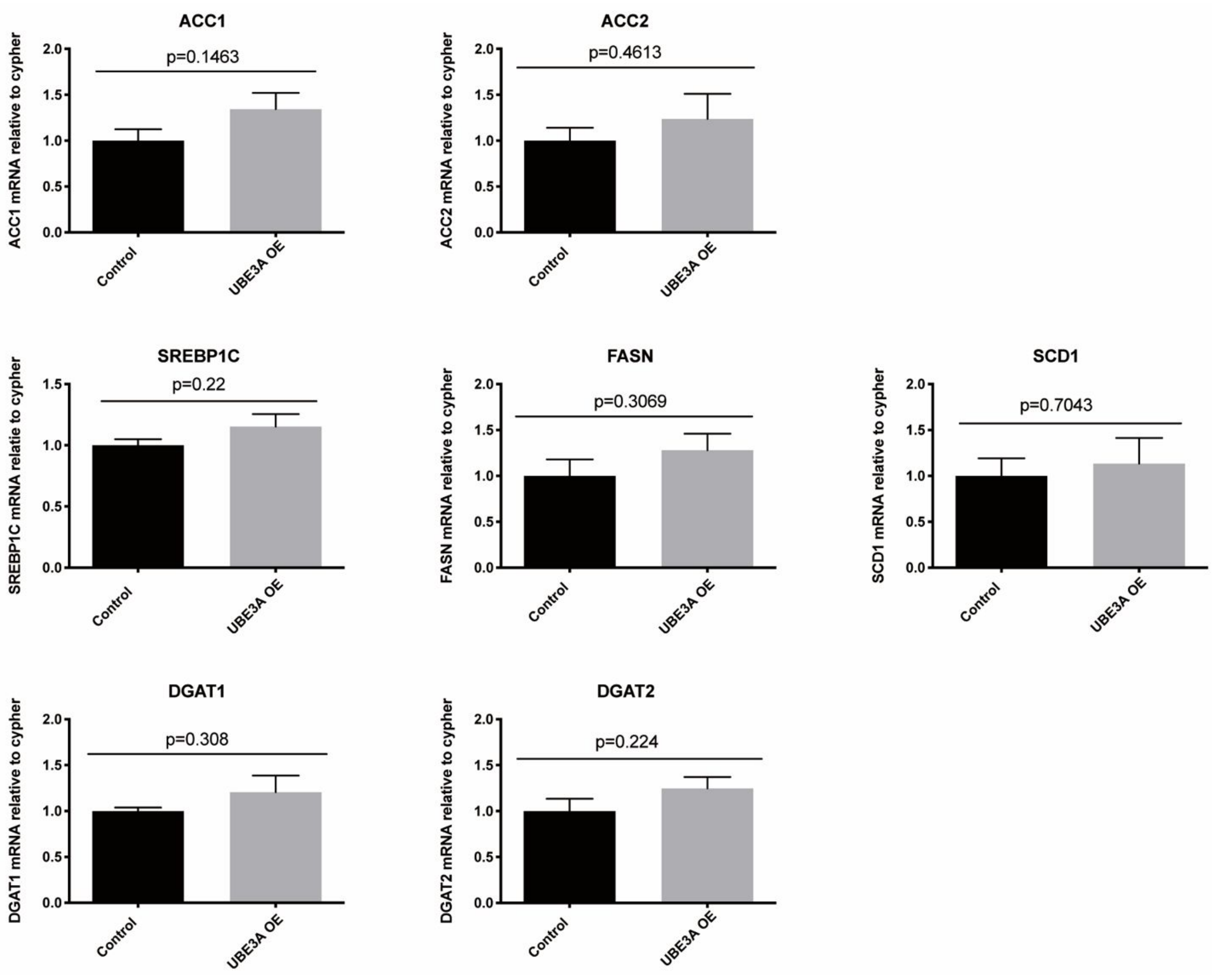

Figure S3. The mRNA levels of lipogenic expression in UBE3A-overexpressing liver. Quantitative RT-PCR analysis revealed no change in the expression of lipogenic genes including ACC1, ACC2, SREBP1C, FASN, SCD1 and TG-synthesizing genes DGAT1, DGAT2 in UBE3A-overexpressing liver compared to the control.
